# EEG Changes Due to Experimentally Induced 3G Mobile Phone Radiation

**DOI:** 10.1371/journal.pone.0129496

**Published:** 2015-06-08

**Authors:** Suzanne Roggeveen, Jim van Os, Wolfgang Viechtbauer, Richel Lousberg

**Affiliations:** 1 Department of Psychiatry and Psychology, Maastricht University, Maastricht, The Netherlands; 2 King’s College London, King’s Health Partners, Department of Psychosis Studies, Institute of Psychiatry, London, United Kingdom; Kagoshima University Graduate School of Medical and Dental Sciences, JAPAN

## Abstract

The aim of this study was to investigate whether a 15-minute placement of a 3G dialing mobile phone causes direct changes in EEG activity compared to the placement of a sham phone. Furthermore, it was investigated whether placement of the mobile phone on the ear or the heart would result in different outcomes. Thirty-one healthy females participated. All subjects were measured twice: on one of the two days the mobile phone was attached to the ear, the other day to the chest. In this single-blind, cross-over design, assessments in the sham phone condition were conducted directly preceding and following the mobile phone exposure. During each assessment, EEG activity and radiofrequency radiation were recorded jointly. Delta, theta, alpha, slowbeta, fastbeta, and gamma activity was computed. The association between radiation exposure and the EEG was tested using multilevel random regression analyses with radiation as predictor of main interest. Significant radiation effects were found for the alpha, slowbeta, fastbeta, and gamma bands. When analyzed separately, ear location of the phone was associated with significant results, while chest placement was not. The results support the notion that EEG alterations are associated with mobile phone usage and that the effect is dependent on site of placement. Further studies are required to demonstrate the physiological relevance of these findings.

## Introduction

Mobile phone usage has become an integrated part of modern society. In recent years, number and level of usage of mobile phones has increased rapidly. In 2013, 6.8 billion mobile phone subscriptions were registered [1]. In recent years, new technology of 3^rd^ generation (3G) or universal mobile telecommunication system (UMTS), using the 1.9–2.1 GHz frequency band has been introduced, followed by the 4^th^ generation. Although there are some worries [2] about the potential negative effects of RF-EMF on health, large sections of the population are avid customers. The number of studies investigating whether or not RF-EMF has adverse health effects has increased rapidly. The research field is broad since several areas are explored, ranging from carcinogenesis and infertility to basic effects on physiological parameters. In general, results are conflicting and inconclusive [3–6]. While a number of studies demonstrate influence of radiation on health, others cannot replicate these findings. Nevertheless, the International Agency for Research on Cancer concluded that there is “limited evidence in humans” and in 2011 it was decided to classify mobile phone radiation in group 2B—possibly carcinogenic [2]. In contrast, the 2012 overview report of the Mobile Telecommunications and Health Research (MTHR) Programme concluded that there is no evidence of carcinogenic effects due to exposure to mobile phone signals [7]. However, examining possible effects due to long-term exposure and the effects on other outcomes were suggested as priority research areas. Also in the WHO Research Agenda for Radiofrequency Fields of 2011, “provocation studies to identify neurobiological mechanisms underlying possible effects of RF on brain function, including sleep and resting EEG” are considered to have a high priority [8].

The effects of mobile phone radiation on electroencephalography (EEG) have been studied since the mid-nineties [9]. There are a number of studies which have investigated the effects of mobile phone radiation on resting EEG, on sleep EEG, on the performance of cognitive tasks, and on event related potentials (ERP) of conscious sensory stimuli. Apart from a relative consistent finding [9–15] of an increased power in the alpha band, no consistent results have been reported. A possible contributing factor to these varying results may be found in the diversity of designs and in the statistical analyses. Some examples are: type of exposure (network 2G/3G and a real mobile phone versus a radiating module), duration of exposure (ranging from minutes to several hours), and statistical tests (parametric versus non-parametric tests). Apart from these methodological aspects, it has been suggested that source of funding might influence the results [16]. It has been shown that 87% of brain activity studies are sponsored by the mobile phone industry [4]. Although this does not necessarily imply that the results of these articles are biased, the issue of conflict of interest cannot be neglected.

To shed further light on this topic, it was decided to set up a basic experiment to investigate whether EEG is influenced by mobile phone radiation. The focus on EEG as the dependent variable was deliberate since functioning of brain tissue is based on electrochemical processes [17] and interference by an electromagnetic device (mobile phone) placed directly against the head is, from a physical point of view, very likely to occur. Despite this plausible physical interference mechanism, adverse effects are rarely reported.

Before setting up the experiment, several methodological issues were considered. First, in most studies a control session was included on a separate day [12–15,18]. It is known, however, that resting EEGs can differ across days, even without any intervention [19]. Therefore, it was decided to compare an exposure condition with two control measurements, directly preceding and following the exposure condition. In addition, a control-exposure-control session was conducted on two different days in order to test whether the location of placement of the mobile phone might be of influence. Apart from the obvious placement of the mobile phone on the ear, it was decided to also place the phone on the chest. Any differences in outcome between placement locations may be informative about a working mechanism. Third, an actual mobile phone was used instead of a GSM module or other method to simulate mobile phone radiation. This decision was made to represent reality as accurately as possible. In order to ensure that the radiation did not have a direct effect on the measurement equipment, shielded EEG electrodes were used. Finally, multilevel random regression analysis was used instead of ANOVA techniques. The main reason is that EEG data show hierarchical clustering in at least four levels: subjects, sessions, conditions and EEG segments. Multilevel regression analysis also allows modulation of random effects (regression coefficients may vary between subjects) as well as the incorporation of an autoregressive covariance structure (since consecutive EEG segments are highly correlated).

Two a priori hypotheses were tested. Based on existing literature, an increase in alpha activity during exposure was expected. Second, because of the smaller distance to the brain, the influence on the EEG of radiation was expected to be larger with ear placement as compared to chest placement.

## Materials and Methods

### Participants

Thirty-one female participants (mean age of 26.7 years; SD = 8.5), non-smoking, and without a medical history of cardiac or nervous system disorders were included. Four hours prior to the start of the session, no caffeine-containing beverages were used. No alcohol was used in the preceding 12 hours and sufficient night rest was ensured. After reading a document with detailed information about the study and having discussed any possible concerns with the researcher, subjects gave their verbal and written informed consent. Complete participation was compensated with €50,-.

### Experimental procedures

The study consisted of two sessions, each session taking place on a separate day, with a maximum of two days in between the two sessions. The experiments were conducted in an electrically non-shielded, room. The sequence of placement on the ear or heart was counterbalanced between the sessions. EEG was measured using shielded electrodes. Each shielded electrode had a separate ground plug, which was connected into a general ground-device. The following EEG electrodes were placed in accordance to the 10–20 system [20]: Fz, F3, F4, Cz, C3, C4, Pz, P3, P4, Oz, O1, and O2. All electrodes were fixed using conductive paste [21]. A reference was placed on each ear lobe. To check for possible eye movements, an electro-oculogram (EOG) electrode was placed 1 centimetre under the midline of both eyes. The electrodes were connected to a BrainAmp amplifier (Brain Products). Impedances were maintained below 5 kΩ. Spike artefacts due to radiation, which are mentioned in other articles [4,22], were not observed in the data. Both EEG and radiation data were sampled with 1000 Hz using Brain Vision Recorder software. Each participant was exposed to four consecutive conditions during each session, according to the schedule shown in Table 1. There were three conditions with a sham phone, and one condition with a dialling mobile phone. The experimenter entered the room at the end of each 15 minute condition to change the phone. During this exchange of phones, no electrophysiological measurements took place. In the case of two consecutive sham phone conditions, the same procedure was followed (a second sham phone was placed). In order to ensure blinding, the order of the conditions was unknown to the participant, thus achieving a single-blind experiment. In one session, the ‘dialling’ condition was in the second quarter of an hour and in the other session the ‘dialling’ condition was in the third quarter. This sequence order was balanced over the subjects. Subjects were not aware of the different phones used.

**Table 1:** Experimental design.

	15 minutes	15 minutes	15 minutes	15 minutes
**Day 1 or 2**	Pre-exposure (PRE)	Exposure (EXP)	Post-exposure (POST)	Not used
**Day 1 or 2**	Not used	Pre-exposure (PRE)	Exposure (EXP)	Post-exposure (POST)

The sequence was randomly determined in order to ensure blinding of the participant. In conditions labelled as ‘not used’, an identical sham phone was placed in the same way as in the pre- and post-exposure conditions.

### Exposure

- A 3G smartphone was used. During exposure conditions, the phone was dialled from a fixed line in another room. No sound was exchanged (mute settings), and vibration mode was off, in order to ensure that the participant could not identify the dialling condition.
- The SAR level of the phone was reported as 0.69 W/kg (head) in the manual.
- The sham phone was a non-functioning replica of the same weight and the same characteristics as the functioning smartphone. The sham phone contained the same type of battery as the real phone and care was taken that the battery was inactive in the sham condition. In a pilot study before the start of the actual experiment, no evidence was found that participants could detect differences between the actual mobile phone and the sham phone. As a check, subjects were asked whether they noticed any differences between phone placements after each session in the experiment.

Radiation activity was detected with a radiation detector (HF59B, Gigahertz Solutions), connected to an omnidirectional antenna. This detector was connected (from the DC output) to the BrainAmp headbox with an auxiliary plug. The detector was placed in the upright position, 30 cm above the table (at which the participant was sitting) and 20 cm left from the participant. In one of the two sessions, the phone/sham-phone was placed directly onto the left ear, ensuring that there was no contact between the phone and the EEG electrodes. The position of the phone was comparable to a typical dialling position, in an angle of approximately 45 degrees. During the other session, the phone was placed adjacent to the left side of the sternum, bordering the sternoclavicular joint. Previous tests showed that there was neither a direct interference of the mobile phone radiation on the shielded electrodes nor on the internal ADC converter of the amplifier. The rear side of the phone was placed on the skin in both sessions. The phone was fixed using an elastic band.

In order to investigate radiation exposure, a Network Analyzer, Agilent Technologies, E5061B ENA Series, 5 Hz—3 GHz was used. The frequency band operated in the following frequency: 1.9291 to 1.9397 GHz. A radiation peak as measured with the radiation detector, equalled a power of approximately 10 dbm measured next to the ear with the Network Analyzer.

In order to maintain the participant’s alertness and to guarantee a relatively stable mood, participants watched an affectively neutral documentary about the development of the earth. All experimental sessions were performed between 09.00 and 17.00 o’clock.

### Data reduction

EEG data was analysed offline with the software program BrainVision Analyzer 2.0 (Brain Products, München, Germany). Data were filtered using a high cut-off filter of 50 Hz and a low cut-off filter of 0.5 Hz. Each measurement was divided in epochs of 32768ms. Subsequently, a fast Fourier transformation (FFT) was executed for each epoch. The following frequency bands were computed: delta ranging from 0.5 to 4.0 Hz, theta (4.0 to 7.5 Hz), alpha (7.5 to 13.0 Hz), slow beta (13.0 to 20.0 Hz), fast beta (20.0 to 30.0 Hz), and gamma (30.0 to 47.0 Hz). Data was exported to SPSS 21.0. The SPSS dataset was constructed in such a way that each record contained the unified information of the EEG bands per 32768 ms. This means that each 15 minute condition was subdivided into 27 consecutive ‘segments’. All EEG variables were log_10_-transformed because of a positively skewed distribution. In order to reduce the total number of 72 EEG dependent variables (12 locations x 6 bands), the locations were bundled into brain regions per frequency band: left—midline—right and frontal—central—parietal—occipital. For example ‘frontal-alpha’ was computed as the average of the (log_10_-transformed) Fz, F3, and F4 alpha activity.

EOG and radiation data were also divided into 32768ms epochs, but no FFT was carried out in this data. The sum of EOG activity was computed for each segment. The same procedure was performed for radiation.

### Statistical analysis

Statistical analyses were performed using SPSS 21.0. Multilevel random regression analyses were used to investigate the effect of radiation on the EEG outcome measures. The 27 consecutive segments within each condition were treated as the repeated measure variable. The multilevel regression analyses contained four levels: subject, session (every subject was measured on two separate days), condition (three conditions within each session), and segment (27 within each condition). As mentioned in the introduction, EEG activity of consecutive segments is strongly interdependent. An autoregressive (AR1) covariance structure was found to be most suitable, for the residuals at the fourth (segment) level. In order to find the optimal covariance structure, likelihood ratio tests were conducted to compare models with different covariance structures (independent, autoregressive and autoregressive moving average structures were compared). This is a well-established approach for selecting amongst various structures [23, 24]. An SPSS syntax example of this model is shown below.

MIXED mean_delta_left WITH PRE POST radiation segment session age ear_heart timing_of_exposure EOGleft EOGright

/CRITERIA = CIN(95)MXITER(100)MXSTEP(10)SCORING(1)SINGULAR(0.000000000001) HCONVERGE(0, ABSOLUTE) LCONVERGE(0, ABSOLUTE) PCONVERGE(0.000001, ABSOLUTE)

/FIXED = radiation session segment EOGleft EOGright ear_heart timing_of_exposure age

PRE POST | SSTYPE(3)

/METHOD = REML

/PRINT = SOLUTION TESTCOV

/RANDOM = INTERCEPT segment | SUBJECT(subject) COVTYPE(VC)

/RANDOM = INTERCEPT segment | SUBJECT(subject *session) COVTYPE(VC)

/RANDOM = INTERCEPT segment | SUBJECT(subject *session*condition) COVTYPE(VC)

/REPEATED = segment | SUBJECT(subject *session* condition) COVTYPE(AR1).

All segments with outlying (above percentile 99) EOG activity were rejected from the analyses (2.3% of all segments). Furthermore, all remaining EOG information (the summed activity per segment) was included in all statistical models as a covariate. The main predictor of interest was the summed radiation for each segment. Furthermore, five design factors were included in the analyses as covariates: condition (PRE, EXP, and POST recoded into two dummy variables), phone placement location (ear or heart), session (day 1 or day 2), segment (added as a quantitative covariate), and the timing of exposure (second or third 15 minute quarter of the experiment, see Table 1). In addition, we controlled for the effects of age in all analyses by including age as an additional covariate. The statistical Benjamini-Hochberg procedure controls the false discovery rate (FDR) and was used in retrospection to correct for multiple testing.

### Ethics statement

Approval was obtained from the medical ethics committee of the Academic Hospital Maastricht, on June, 6th, 2013.

## Results

After each session, subjects were asked if they noticed any temperature change, or other difference between the conditions. None of the participants could identify noticeable differences.

Table 2 shows the mean spectral power densities of the mean midline (average of Fz, Cz, Pz, and Oz) activity per condition. As can be seen from the raw data in this table, there is a marginally elevated activity during EXP. Considering the standard errors, this elevation is clearly not significant.

**Table 2:** Mean values in the three conditions.

	Mean midline (Fz, Cz, Pz and Oz)								
	PRE (sham)			EXP (dialing phone)			POST (sham)		
	mean	SD	SE	mean	SD	SE	mean	SD	SE
Delta (.5–4 Hz)	1.93	0.18	0.03	1.94	0.18	0.03	1.93	0.18	0.03
Theta (4–7.5 Hz)	1.42	0.16	0.03	1.42	0.16	0.03	1.42	0.17	0.03
Alpha (7.5–13 Hz)	1.22	0.27	0.05	1.24	0.28	0.05	1.23	0.28	0.05
Slow-beta (13–20 Hz)	0.86	0.21	0.04	0.87	0.21	0.04	0.86	0.21	0.04
Fast-beta (20–30 Hz)	0.65	0.18	0.03	0.67	0.18	0.03	0.67	0.19	0.03
Gamma (30–47 Hz)	0.42	0.18	0.03	0.44	0.20	0.04	0.43	0.20	0.04

Mean spectral power densities (μV^2^) in the six frequency bands for PRE, EXP and POST (standard deviation SD and standard error SE are given).

Next, multilevel random regression analyses were performed to test whether an influence of radiation existed on EEG activity, while controlling for the abovementioned series of covariates.

**Fig 1:**
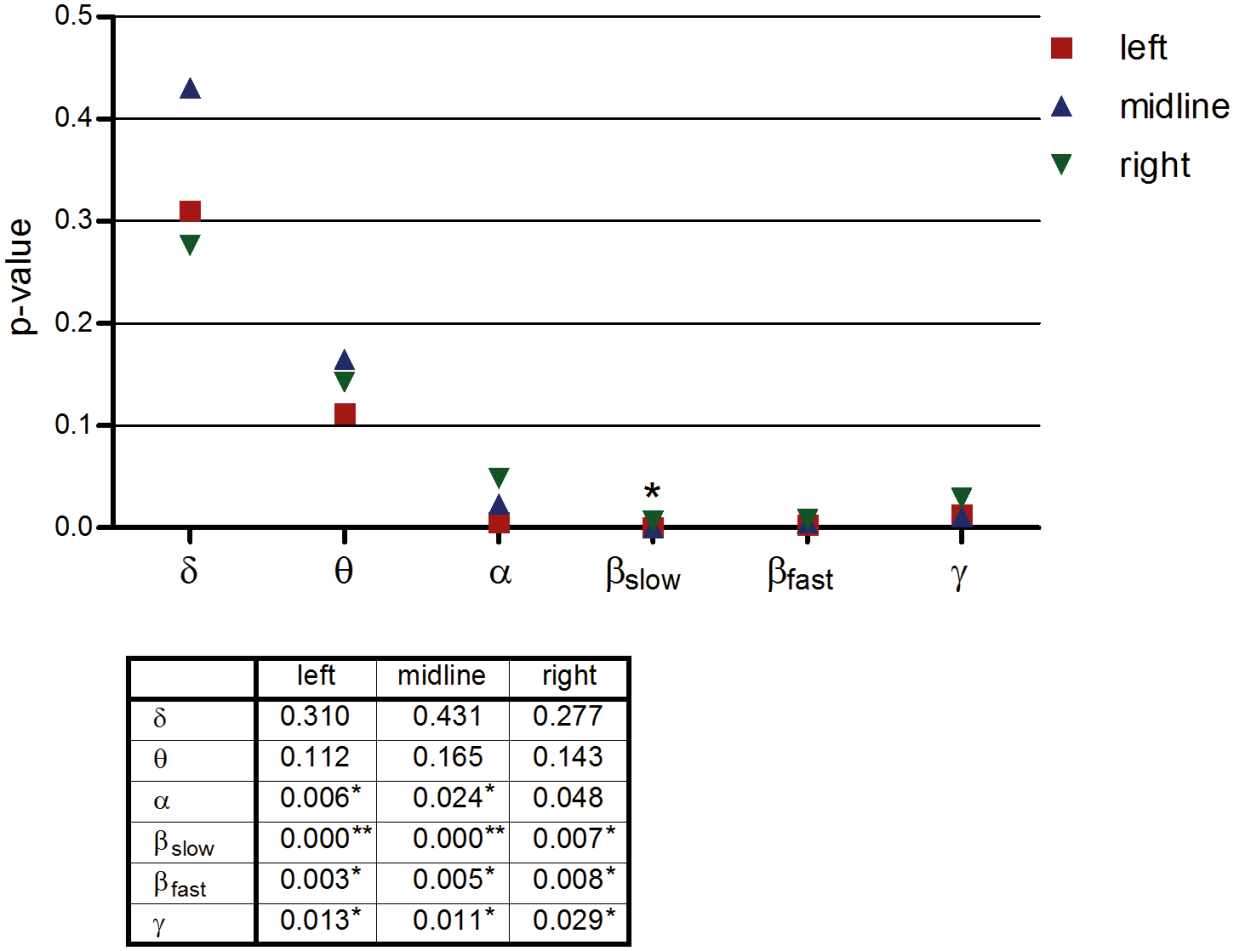
Left midline right. Each symbol represents the p-value of the main predictor ‘radiation’ in the relevant area. * in the graph indicates a significant left-right contrast. Below the graph a table depicts the p-values. * in the table represents a significant q-value (q<0.05) after correction for multiple testing using the false discovery rate (FDR) method. ** significant q-value (q<0.01) after correction for multiple testing. All values of this table were included for the FDR computation.

Fig 1 demonstrates the p-values of the predictor of main interest (radiation) in the eighteen (six EEG bands × three areas) analyses. The three areas refer to the left side of the brain (F3, C3, P3, O1), the midline (Fz, Cz, Pz, Oz), and the right side (F4, C4, P4, O2). All estimates were positive, indicating that an increase in radiation is associated with an increase of EEG band power. The alpha, slow-beta, fast-beta, and gamma bands have significant p-values and q-values. Post-hoc left-right contrasts were tested. Slow beta activity showed a significant left-right difference (P = 0.01), in which the estimates in the left hemisphere were more positive than right. With respect to the covariates, segment had significant p-values in sixteen of the eighteen analyses, especially in the slow beta band (p-values <0.001, left and midline: q-values<0.01). There was an increasing trend within the conditions. In addition, EOG proved, as expected, to be a significantly related to EEG in all analyses (p-values <0.002).

The AR1 covariance structure was justified since all AR1 estimates were highly significant (all Wald Z values > 4 for the rho estimates). Random intercepts were also significant for all models (Wald Z > 3). The random slope for segment was significant (Wald Z > 2) for all frequency bands, except delta. The latter means that each person, in each session and condition has a significantly unique time course of EEG activity (segment representing time).

**Fig 2:**
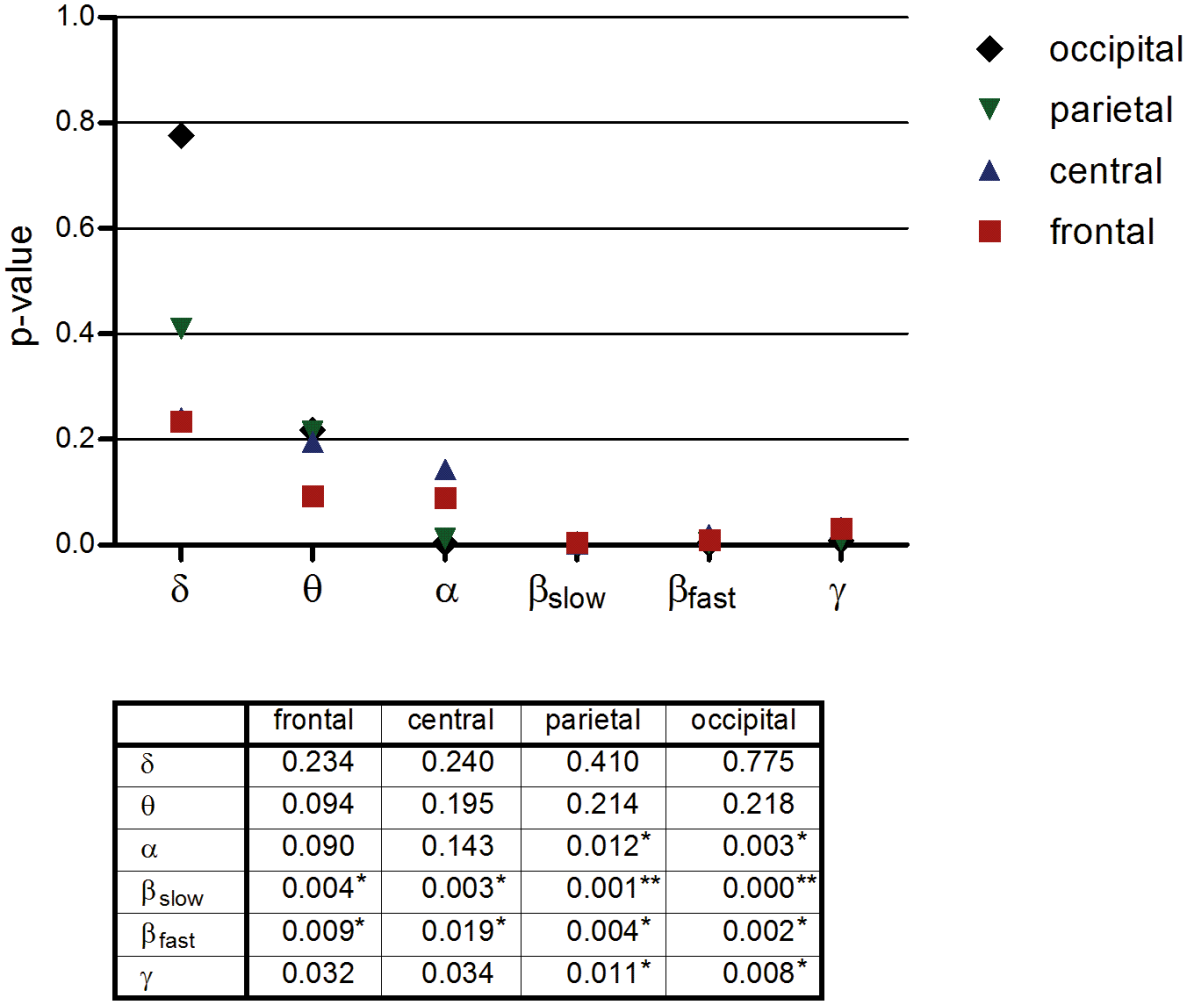
Cortical regions. Each symbol represents the p-value of the main predictor ‘radiation’ in the relevant area. Below the graph a table depicts the p-values. * significant q-value (q<0.05) after correction for multiple testing using the false discovery rate (FDR) method. ** significant q-value (q<0.01) after correction for multiple testing. All values of this table were included for the FDR computation.

Similar analyses were performed, this time for the four cortical regions: frontal (Fz, F3, F4), central (Cz, C3, C4), parietal (Pz, P3, P4), and occipital (Oz, O1, O2). Significant radiation effects were found in the alpha, slowbeta, fastbeta, and gamma bands (Fig 2). With respect to the covariates and random effects, comparable results were obtained as reported in the previous set of analyses.

**Fig 3:**
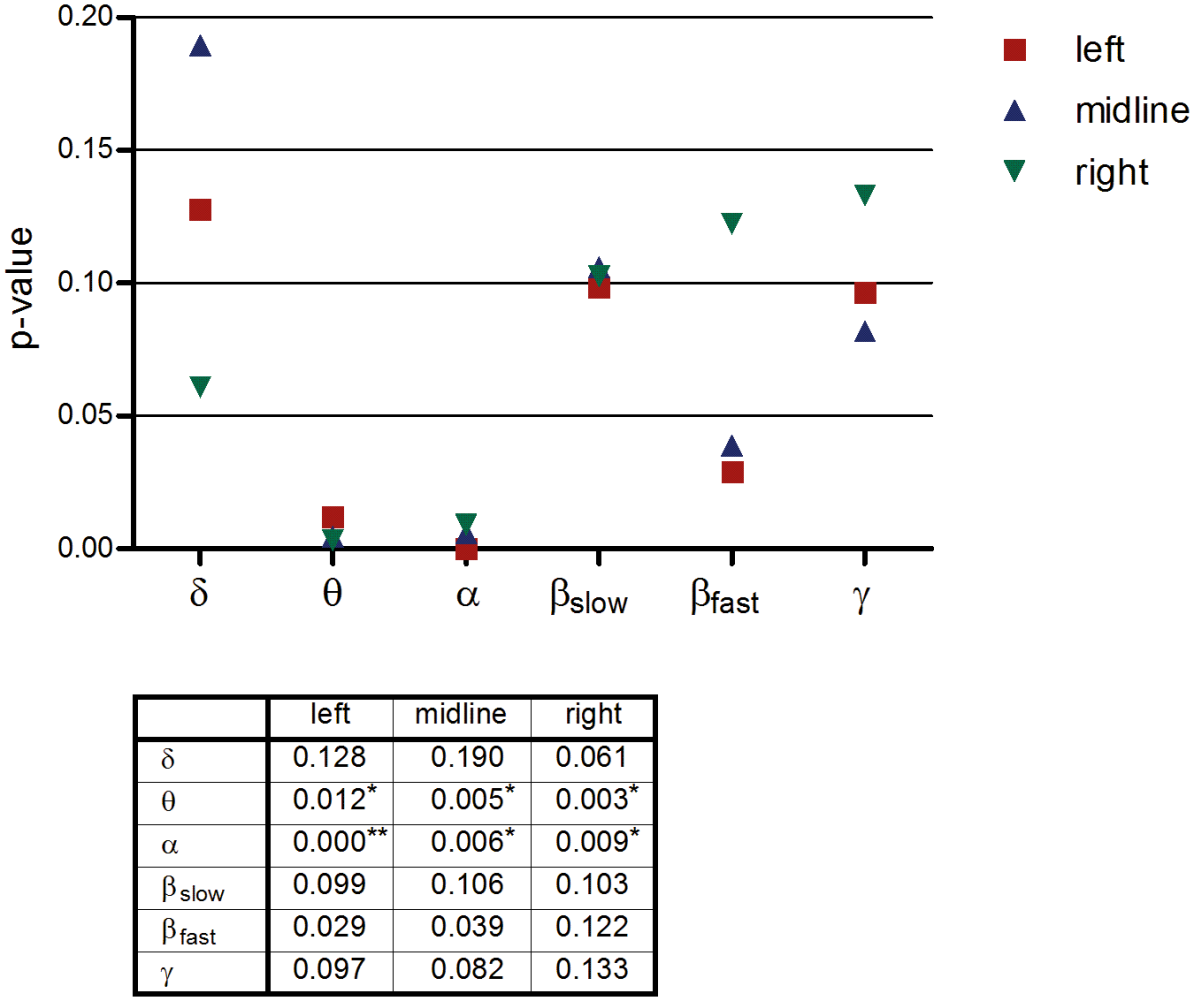
Interaction variable placement*radiation. Each symbol represents the p-value of the interaction variable in the relevant area. Below the graph a table depicts the p-values. * significant q-value (q<0.05) after correction for multiple testing using the false discovery rate (FDR) method. ** significant q-value (q<0.01) after correction for multiple testing. All values of this table were included for the FDR computation.

In the analyses above, the radiation effect was controlled for a main (fixed) effect of location of placement. To further investigate the influence of placement, an interaction between radiation and location of placement was incorporated in the model of the left—midline—right analyses. Fig 3 depicts the p-values of this placement*radiation effect. The theta and alpha range show statistical significance of the interaction. All other interactions, except for fastbeta—left, were not significant. The estimates were negative, which has to be interpreted as a lower effect of radiation on EEG power for the chest placement compared to the ear.

**Fig 4:**
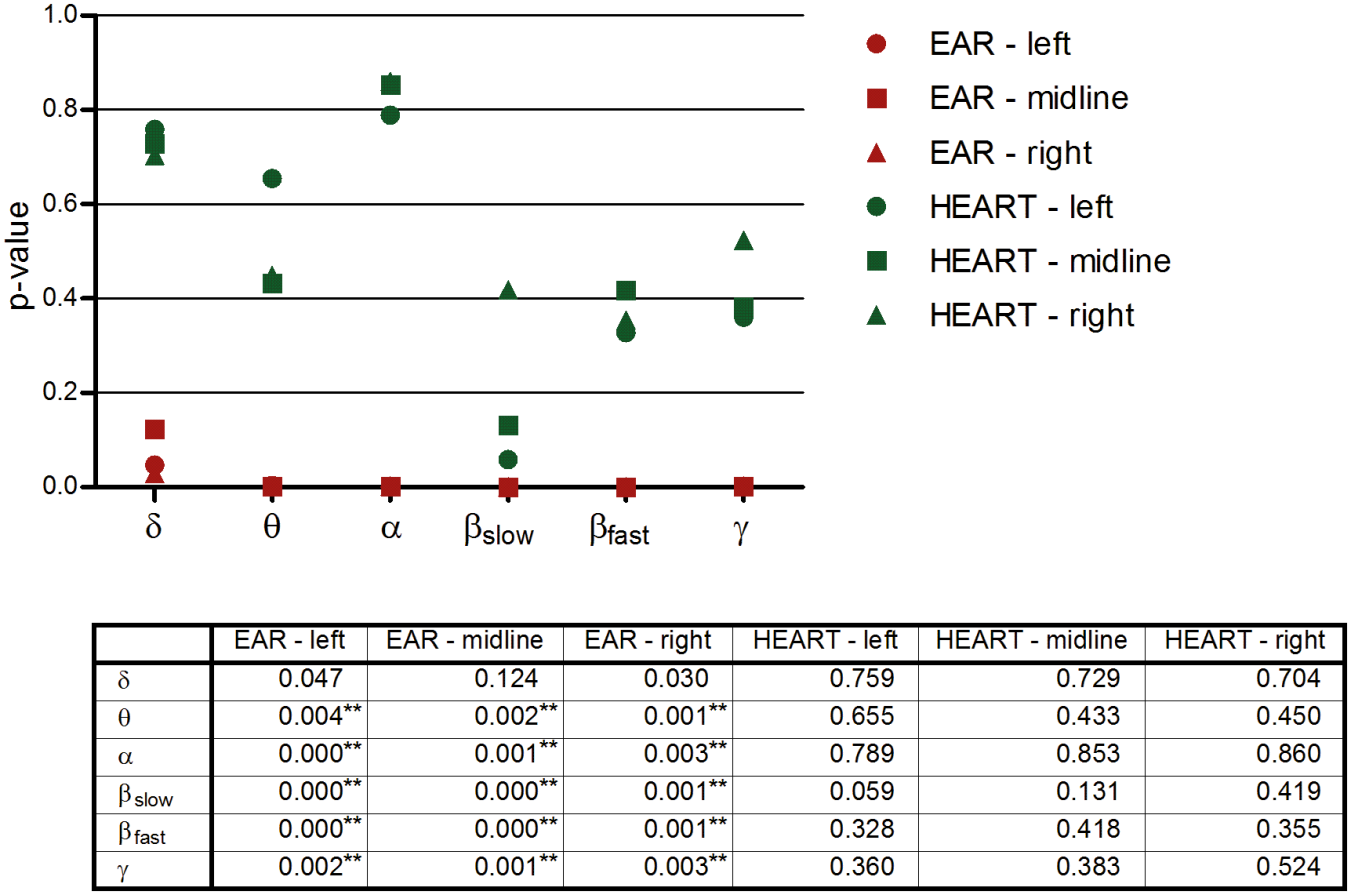
Radiation effects on EEG: ear and heart sessions analyzed separately. Each symbol represents the p-value of the main predictor ‘radiation’ in the relevant area. Below the graph a table depicts the p-values. * significant q-value (q<0.05) after correction for multiple testing using the false discovery rate (FDR) method. ** significant q-value (q<0.01) after correction for multiple testing. All values of this table were included for the FDR computation.

The main radiation effect was analyzed again for ear and heart sessions separately (Fig 4). None of the analyses which were performed on the heart placement data showed a significant outcome, while almost all (except for delta) analyses that were performed on the ear placement data were significant. The interaction effect on the theta and alpha band are evident in this graph.

## Discussion

In this placebo-controlled, single-blind, cross-over study, we investigated whether a 15 minute RF-EMF exposure by a 3G mobile phone impacts EEG activity. It was demonstrated that the results (and conclusions) strongly depend on the method of analysis. Analysed in the traditional fashion, that is, not controlling for the nested structure of the data, no significant differences could be demonstrated (see Table 2), a finding which is in accordance with several other studies [18,25–28]. However, when the data were analysed with an appropriate multilevel statistical model, ‘radiation’ proved to be a significant predictor of the alpha, slow beta, fast beta, and gamma frequency bands over almost all brain regions (Figs 1 and 2). There is a trend for the radiation effect to be stronger on the ipsilateral side. The positive significant time effect (segment) within conditions, observed in all regions and frequency bands, was unexpected and an explanation is lacking at this moment. Guided by the significant placement*radiation interactions, separate analyses for the ear and heart placements made clear that the radiation effect was mainly present in sessions where the mobile phone was placed on the ear (Figs 3 and 4). To our knowledge, this ear-heart contrast has not been studied before. The present data suggest that a greater distance to the brain in case of the chest placement results in less EEG effects.

Although there are some studies which found an increase in the alpha frequency band due to RF-EMF [9–15], the extent to which the different EEG bands are affected in the present study has not been reported in literature to date. This discrepancy needs clarification and probably has to be sought in methodological differences with other studies. As mentioned above, the type of statistical approach is an important factor in this issue. The multilevel approach is the preferred approach given the present study design. As stated in the introduction, this approach allows for modelling clustered data, to correct for autocorrelation, and for modelling random effects. In addition, in this study radiation was used as the predictor of primary interest instead of a general condition (exposed vs. non-exposed conditions) effect. This was made possible by connecting the radiation detector to the amplifier. Another methodological difference may lie in the usage of shielded electrodes (most articles do not describe this specific information) which prevents a direct influence of RF-EMF on the wires. Yet another difference is that in the present study an actual mobile phone was used functioning within the UMTS bandwidth (instead of 2G). The studies which also used UMTS do not find EEG effects [18,27,29]. A final difference pertains to the control measurement. Whereas most studies had the control session on a separate day, the present study used a crossover moment in the same session, directly preceding and following the exposure session.

Some limitations have to be considered. First, the study was performed with adult female subjects only. The generalizability of the results to, for example, male subjects, children, and the elderly has to be demonstrated in future research. Second, a sample size of thirty-one is relatively small. Future studies with larger sample sizes are required. Two other critical points are exposure time and follow up measurements. In this study, only one exposure condition (15 minutes) was used and a (long term) follow up measurement was not included. It would be interesting to investigate what the effects are of other (shorter or longer) exposure periods to mobile phone radiation, as well as to find out what the effects are of frequent experimental exposure to radiation in the long term. Fifth, no inner ear temperature was measured. As there is evidence that mobile phones cause a thermal, heating effect [30], it could be argued that inner ear temperature fluctuations between the conditions may have confounded the findings. On the other hand, it has also been reported that any changes in cerebral blood flow due to mobile phone radiation, assessed by positron emission tomography, are unlikely to be temperature-related [31,32]. This issue needs further investigation. Sixth, the experiment was performed in a non-shielded room, thus including environmental background radiation. It would be ideal to carry out such experiments in a radiation-free environment. As a seventh point of concern, in retrospect it would have been preferable to not enter the experimenting room in-between conditions to change phones. Ideally the phone should be programmed from outside the room. Furthermore, a note should be made with respect to the number of tests performed. For example, Fig 1 contains eighteen test results. When corrected with the Bonferroni procedure, only two p-values (slow-beta left and midline) would remain significant. However, at least some of the findings would hold up under such a correction and it should be noted that the Bonferroni method is actually overly conservative for multiple correlated tests. [33] Instead, the ‘False discovery rate’ (FDR) was used to correct for multiple testing. Most results were still significant after correction. Finally, information on other (psycho)physiological and biological measures may be included in future work.

The question is whether the (temporary) EEG changes, induced by mobile phone radiation, have clinical/adverse consequences. Answering this question is complex and beyond the scope of the present manuscript. First, it is unknown whether mobile phones change EEG activity in the long term. Second, EEG is a reflection of very complex cerebral processes. It is thought that the activity in the different frequency bands represents underlying cortical functions. An example is the thalamocortical network, which plays an important role in the generation of alpha activity [34]. Beta activity, however, only plays a role in the cortex and can, for example, be related to active concentration [35]. Since the functional role of the different frequency bands is still not fully understood, it is also hard to draw conclusions on the (clinical) implications of EEG changes.

In future studies other indicators of brain activity may be included. For example, transcranial magnetic stimulation is a method to test brain excitability. There are several indications that brain excitability is modified due to mobile phone radiation [36–38].

This study attempted to approach the question whether or not mobile phones cause (short-term) changes in EEG activity. There was evidence that mobile phone radiation is associated with increased activity of the alpha, beta, and gamma frequency bands in nearly every brain region. The distance of the mobile phone to the brain was relevant, a larger distance resulting in less or no EEG interference. Replication of the present findings and investigation of possible long term (clinically relevant) effects is urgently required.

## Supporting Information

S1 Excel Datafile. (XLS)

S1 Text SPSS syntax. (DOCX)

S2 Text SPSS syntax. (DOCX)
