# Supplementary material for: EEG Changes Due to Experimentally Induced 3G Mobile Phone Radiation
Source: PLoS One. 2015 Jun 8;10(6):e0129496. doi: 10.1371/journal.pone.0129496 (PMC4459698; doi:10.1371/journal.pone.0129496)
Supplement: S1 Text — (DOCX) [file pone.0129496.s002.docx]

USE ALL.

COMPUTE filter_$=(EOGlinks_sum_mean < 10000000 and EOGrechts_sum_mean < 10000000 and straling_valide = 1).

FILTER BY filter_$.

EXECUTE.

MIXED mean_delta_links WITH PRE POST segment meting leeftijd oor_hart moment_exposure

straling EOGlinks EOGrechts

/CRITERIA=CIN(95) MXITER(100) MXSTEP(10) SCORING(1) SINGULAR(0.000000000001) HCONVERGE(0,

ABSOLUTE) LCONVERGE(0, ABSOLUTE) PCONVERGE(0.000001, ABSOLUTE)

/FIXED= leeftijd EOGlinks EOGrechts

oor_hart moment_exposure meting straling segment

PRE POST | SSTYPE(3)

/METHOD=REML

/PRINT=SOLUTION TESTCOV

/RANDOM=INTERCEPT segment | SUBJECT(ppn) COVTYPE(VC)

/RANDOM=INTERCEPT segment | SUBJECT(ppn*meting) COVTYPE(VC)

/RANDOM=INTERCEPT segment | SUBJECT(ppn*meting*conditie) COVTYPE(VC)

/REPEATED=segment | SUBJECT(ppn*meting*conditie) COVTYPE(AR1).

MIXED mean_delta_midden WITH PRE POST segment meting leeftijd oor_hart moment_exposure

straling EOGlinks EOGrechts

/CRITERIA=CIN(95) MXITER(100) MXSTEP(10) SCORING(1) SINGULAR(0.000000000001) HCONVERGE(0,

ABSOLUTE) LCONVERGE(0, ABSOLUTE) PCONVERGE(0.000001, ABSOLUTE)

/FIXED= leeftijd EOGlinks EOGrechts

oor_hart moment_exposure meting straling segment

PRE POST | SSTYPE(3)

/METHOD=REML

/PRINT=SOLUTION TESTCOV

/RANDOM=INTERCEPT segment | SUBJECT(ppn) COVTYPE(VC)

/RANDOM=INTERCEPT segment | SUBJECT(ppn*meting) COVTYPE(VC)

/RANDOM=INTERCEPT segment | SUBJECT(ppn*meting*conditie) COVTYPE(VC)

/REPEATED=segment | SUBJECT(ppn*meting*conditie) COVTYPE(AR1).

MIXED mean_delta_rechts WITH PRE POST segment meting leeftijd oor_hart moment_exposure

straling EOGlinks EOGrechts

/CRITERIA=CIN(95) MXITER(100) MXSTEP(10) SCORING(1) SINGULAR(0.000000000001) HCONVERGE(0,

ABSOLUTE) LCONVERGE(0, ABSOLUTE) PCONVERGE(0.000001, ABSOLUTE)

/FIXED= leeftijd EOGlinks EOGrechts

oor_hart moment_exposure meting straling segment

PRE POST | SSTYPE(3)

/METHOD=REML

/PRINT=SOLUTION TESTCOV

/RANDOM=INTERCEPT segment | SUBJECT(ppn) COVTYPE(VC)

/RANDOM=INTERCEPT segment | SUBJECT(ppn*meting) COVTYPE(VC)

/RANDOM=INTERCEPT segment | SUBJECT(ppn*meting*conditie) COVTYPE(VC)

/REPEATED=segment | SUBJECT(ppn*meting*conditie) COVTYPE(AR1).

MIXED mean_theta_links WITH PRE POST segment meting leeftijd oor_hart moment_exposure

straling EOGlinks EOGrechts

/CRITERIA=CIN(95) MXITER(100) MXSTEP(10) SCORING(1) SINGULAR(0.000000000001) HCONVERGE(0,

ABSOLUTE) LCONVERGE(0, ABSOLUTE) PCONVERGE(0.000001, ABSOLUTE)

/FIXED= leeftijd EOGlinks EOGrechts

oor_hart moment_exposure meting straling segment

PRE POST | SSTYPE(3)

/METHOD=REML

/PRINT=SOLUTION TESTCOV

/RANDOM=INTERCEPT segment | SUBJECT(ppn) COVTYPE(VC)

/RANDOM=INTERCEPT segment | SUBJECT(ppn*meting) COVTYPE(VC)

/RANDOM=INTERCEPT segment | SUBJECT(ppn*meting*conditie) COVTYPE(VC)

/REPEATED=segment | SUBJECT(ppn*meting*conditie) COVTYPE(AR1).

MIXED mean_theta_midden WITH PRE POST segment meting leeftijd oor_hart moment_exposure

straling EOGlinks EOGrechts

/CRITERIA=CIN(95) MXITER(100) MXSTEP(10) SCORING(1) SINGULAR(0.000000000001) HCONVERGE(0,

ABSOLUTE) LCONVERGE(0, ABSOLUTE) PCONVERGE(0.000001, ABSOLUTE)

/FIXED= leeftijd EOGlinks EOGrechts

oor_hart moment_exposure meting straling segment

PRE POST | SSTYPE(3)

/METHOD=REML

/PRINT=SOLUTION TESTCOV

/RANDOM=INTERCEPT segment | SUBJECT(ppn) COVTYPE(VC)

/RANDOM=INTERCEPT segment | SUBJECT(ppn*meting) COVTYPE(VC)

/RANDOM=INTERCEPT segment | SUBJECT(ppn*meting*conditie) COVTYPE(VC)

/REPEATED=segment | SUBJECT(ppn*meting*conditie) COVTYPE(AR1).

MIXED mean_theta_rechts WITH PRE POST segment meting leeftijd oor_hart moment_exposure

straling EOGlinks EOGrechts

/CRITERIA=CIN(95) MXITER(100) MXSTEP(10) SCORING(1) SINGULAR(0.000000000001) HCONVERGE(0,

ABSOLUTE) LCONVERGE(0, ABSOLUTE) PCONVERGE(0.000001, ABSOLUTE)

/FIXED= leeftijd EOGlinks EOGrechts

oor_hart moment_exposure meting straling segment

PRE POST | SSTYPE(3)

/METHOD=REML

/PRINT=SOLUTION TESTCOV

/RANDOM=INTERCEPT segment | SUBJECT(ppn) COVTYPE(VC)

/RANDOM=INTERCEPT segment | SUBJECT(ppn*meting) COVTYPE(VC)

/RANDOM=INTERCEPT segment | SUBJECT(ppn*meting*conditie) COVTYPE(VC)

/REPEATED=segment | SUBJECT(ppn*meting*conditie) COVTYPE(AR1).

MIXED mean_alpha_links WITH PRE POST segment meting leeftijd oor_hart moment_exposure

straling EOGlinks EOGrechts

/CRITERIA=CIN(95) MXITER(100) MXSTEP(10) SCORING(1) SINGULAR(0.000000000001) HCONVERGE(0,

ABSOLUTE) LCONVERGE(0, ABSOLUTE) PCONVERGE(0.000001, ABSOLUTE)

/FIXED= leeftijd EOGlinks EOGrechts

oor_hart moment_exposure meting straling segment

PRE POST | SSTYPE(3)

/METHOD=REML

/PRINT=SOLUTION TESTCOV

/RANDOM=INTERCEPT segment | SUBJECT(ppn) COVTYPE(VC)

/RANDOM=INTERCEPT segment | SUBJECT(ppn*meting) COVTYPE(VC)

/RANDOM=INTERCEPT segment | SUBJECT(ppn*meting*conditie) COVTYPE(VC)

/REPEATED=segment | SUBJECT(ppn*meting*conditie) COVTYPE(AR1).

MIXED mean_alpha_midden WITH PRE POST segment meting leeftijd oor_hart moment_exposure

straling EOGlinks EOGrechts

/CRITERIA=CIN(95) MXITER(100) MXSTEP(10) SCORING(1) SINGULAR(0.000000000001) HCONVERGE(0,

ABSOLUTE) LCONVERGE(0, ABSOLUTE) PCONVERGE(0.000001, ABSOLUTE)

/FIXED= leeftijd EOGlinks EOGrechts

oor_hart moment_exposure meting straling segment

PRE POST | SSTYPE(3)

/METHOD=REML

/PRINT=SOLUTION TESTCOV

/RANDOM=INTERCEPT segment | SUBJECT(ppn) COVTYPE(VC)

/RANDOM=INTERCEPT segment | SUBJECT(ppn*meting) COVTYPE(VC)

/RANDOM=INTERCEPT segment | SUBJECT(ppn*meting*conditie) COVTYPE(VC)

/REPEATED=segment | SUBJECT(ppn*meting*conditie) COVTYPE(AR1).

MIXED mean_alpha_rechts WITH PRE POST segment meting leeftijd oor_hart moment_exposure

straling EOGlinks EOGrechts

/CRITERIA=CIN(95) MXITER(100) MXSTEP(10) SCORING(1) SINGULAR(0.000000000001) HCONVERGE(0,

ABSOLUTE) LCONVERGE(0, ABSOLUTE) PCONVERGE(0.000001, ABSOLUTE)

/FIXED= leeftijd EOGlinks EOGrechts

oor_hart moment_exposure meting straling segment

PRE POST | SSTYPE(3)

/METHOD=REML

/PRINT=SOLUTION TESTCOV

/RANDOM=INTERCEPT segment | SUBJECT(ppn) COVTYPE(VC)

/RANDOM=INTERCEPT segment | SUBJECT(ppn*meting) COVTYPE(VC)

/RANDOM=INTERCEPT segment | SUBJECT(ppn*meting*conditie) COVTYPE(VC)

/REPEATED=segment | SUBJECT(ppn*meting*conditie) COVTYPE(AR1).

MIXED mean_slowbeta_links WITH PRE POST segment meting leeftijd oor_hart moment_exposure

straling EOGlinks EOGrechts

/CRITERIA=CIN(95) MXITER(100) MXSTEP(10) SCORING(1) SINGULAR(0.000000000001) HCONVERGE(0,

ABSOLUTE) LCONVERGE(0, ABSOLUTE) PCONVERGE(0.000001, ABSOLUTE)

/FIXED= leeftijd EOGlinks EOGrechts

oor_hart moment_exposure meting straling segment

PRE POST | SSTYPE(3)

/METHOD=REML

/PRINT=SOLUTION TESTCOV

/RANDOM=INTERCEPT segment | SUBJECT(ppn) COVTYPE(VC)

/RANDOM=INTERCEPT segment | SUBJECT(ppn*meting) COVTYPE(VC)

/RANDOM=INTERCEPT segment | SUBJECT(ppn*meting*conditie) COVTYPE(VC)

/REPEATED=segment | SUBJECT(ppn*meting*conditie) COVTYPE(AR1).

MIXED mean_slowbeta_midden WITH PRE POST segment meting leeftijd oor_hart moment_exposure

straling EOGlinks EOGrechts

/CRITERIA=CIN(95) MXITER(100) MXSTEP(10) SCORING(1) SINGULAR(0.000000000001) HCONVERGE(0,

ABSOLUTE) LCONVERGE(0, ABSOLUTE) PCONVERGE(0.000001, ABSOLUTE)

/FIXED= leeftijd EOGlinks EOGrechts

oor_hart moment_exposure meting straling segment

PRE POST | SSTYPE(3)

/METHOD=REML

/PRINT=SOLUTION TESTCOV

/RANDOM=INTERCEPT segment | SUBJECT(ppn) COVTYPE(VC)

/RANDOM=INTERCEPT segment | SUBJECT(ppn*meting) COVTYPE(VC)

/RANDOM=INTERCEPT segment | SUBJECT(ppn*meting*conditie) COVTYPE(VC)

/REPEATED=segment | SUBJECT(ppn*meting*conditie) COVTYPE(AR1).

MIXED mean_slowbeta_rechts WITH PRE POST segment meting leeftijd oor_hart moment_exposure

straling EOGlinks EOGrechts

/CRITERIA=CIN(95) MXITER(100) MXSTEP(10) SCORING(1) SINGULAR(0.000000000001) HCONVERGE(0,

ABSOLUTE) LCONVERGE(0, ABSOLUTE) PCONVERGE(0.000001, ABSOLUTE)

/FIXED= leeftijd EOGlinks EOGrechts

oor_hart moment_exposure meting straling segment

PRE POST | SSTYPE(3)

/METHOD=REML

/PRINT=SOLUTION TESTCOV

/RANDOM=INTERCEPT segment | SUBJECT(ppn) COVTYPE(VC)

/RANDOM=INTERCEPT segment | SUBJECT(ppn*meting) COVTYPE(VC)

/RANDOM=INTERCEPT segment | SUBJECT(ppn*meting*conditie) COVTYPE(VC)

/REPEATED=segment | SUBJECT(ppn*meting*conditie) COVTYPE(AR1).

MIXED mean_fastbeta_links WITH PRE POST segment meting leeftijd oor_hart moment_exposure

straling EOGlinks EOGrechts

/CRITERIA=CIN(95) MXITER(100) MXSTEP(10) SCORING(1) SINGULAR(0.000000000001) HCONVERGE(0,

ABSOLUTE) LCONVERGE(0, ABSOLUTE) PCONVERGE(0.000001, ABSOLUTE)

/FIXED= leeftijd EOGlinks EOGrechts

oor_hart moment_exposure meting straling segment

PRE POST | SSTYPE(3)

/METHOD=REML

/PRINT=SOLUTION TESTCOV

/RANDOM=INTERCEPT segment | SUBJECT(ppn) COVTYPE(VC)

/RANDOM=INTERCEPT segment | SUBJECT(ppn*meting) COVTYPE(VC)

/RANDOM=INTERCEPT segment | SUBJECT(ppn*meting*conditie) COVTYPE(VC)

/REPEATED=segment | SUBJECT(ppn*meting*conditie) COVTYPE(AR1).

MIXED mean_fastbeta_midden WITH PRE POST segment meting leeftijd oor_hart moment_exposure

straling EOGlinks EOGrechts

/CRITERIA=CIN(95) MXITER(100) MXSTEP(10) SCORING(1) SINGULAR(0.000000000001) HCONVERGE(0,

ABSOLUTE) LCONVERGE(0, ABSOLUTE) PCONVERGE(0.000001, ABSOLUTE)

/FIXED= leeftijd EOGlinks EOGrechts

oor_hart moment_exposure meting straling segment

PRE POST | SSTYPE(3)

/METHOD=REML

/PRINT=SOLUTION TESTCOV

/RANDOM=INTERCEPT segment | SUBJECT(ppn) COVTYPE(VC)

/RANDOM=INTERCEPT segment | SUBJECT(ppn*meting) COVTYPE(VC)

/RANDOM=INTERCEPT segment | SUBJECT(ppn*meting*conditie) COVTYPE(VC)

/REPEATED=segment | SUBJECT(ppn*meting*conditie) COVTYPE(AR1).

MIXED mean_fastbeta_rechts WITH PRE POST segment meting leeftijd oor_hart moment_exposure

straling EOGlinks EOGrechts

/CRITERIA=CIN(95) MXITER(100) MXSTEP(10) SCORING(1) SINGULAR(0.000000000001) HCONVERGE(0,

ABSOLUTE) LCONVERGE(0, ABSOLUTE) PCONVERGE(0.000001, ABSOLUTE)

/FIXED= leeftijd EOGlinks EOGrechts

oor_hart moment_exposure meting straling segment

PRE POST | SSTYPE(3)

/METHOD=REML

/PRINT=SOLUTION TESTCOV

/RANDOM=INTERCEPT segment | SUBJECT(ppn) COVTYPE(VC)

/RANDOM=INTERCEPT segment | SUBJECT(ppn*meting) COVTYPE(VC)

/RANDOM=INTERCEPT segment | SUBJECT(ppn*meting*conditie) COVTYPE(VC)

/REPEATED=segment | SUBJECT(ppn*meting*conditie) COVTYPE(AR1).

MIXED mean_gamma_links WITH PRE POST segment meting leeftijd oor_hart moment_exposure

straling EOGlinks EOGrechts

/CRITERIA=CIN(95) MXITER(100) MXSTEP(10) SCORING(1) SINGULAR(0.000000000001) HCONVERGE(0,

ABSOLUTE) LCONVERGE(0, ABSOLUTE) PCONVERGE(0.000001, ABSOLUTE)

/FIXED= leeftijd EOGlinks EOGrechts

oor_hart moment_exposure meting straling segment

PRE POST | SSTYPE(3)

/METHOD=REML

/PRINT=SOLUTION TESTCOV

/RANDOM=INTERCEPT segment | SUBJECT(ppn) COVTYPE(VC)

/RANDOM=INTERCEPT segment | SUBJECT(ppn*meting) COVTYPE(VC)

/RANDOM=INTERCEPT segment | SUBJECT(ppn*meting*conditie) COVTYPE(VC)

/REPEATED=segment | SUBJECT(ppn*meting*conditie) COVTYPE(AR1).

MIXED mean_gamma_midden WITH PRE POST segment meting leeftijd oor_hart moment_exposure

straling EOGlinks EOGrechts

/CRITERIA=CIN(95) MXITER(100) MXSTEP(10) SCORING(1) SINGULAR(0.000000000001) HCONVERGE(0,

ABSOLUTE) LCONVERGE(0, ABSOLUTE) PCONVERGE(0.000001, ABSOLUTE)

/FIXED= leeftijd EOGlinks EOGrechts

oor_hart moment_exposure meting straling segment

PRE POST | SSTYPE(3)

/METHOD=REML

/PRINT=SOLUTION TESTCOV

/RANDOM=INTERCEPT segment | SUBJECT(ppn) COVTYPE(VC)

/RANDOM=INTERCEPT segment | SUBJECT(ppn*meting) COVTYPE(VC)

/RANDOM=INTERCEPT segment | SUBJECT(ppn*meting*conditie) COVTYPE(VC)

/REPEATED=segment | SUBJECT(ppn*meting*conditie) COVTYPE(AR1).

MIXED mean_gamma_rechts WITH PRE POST segment meting leeftijd oor_hart moment_exposure

straling EOGlinks EOGrechts

/CRITERIA=CIN(95) MXITER(100) MXSTEP(10) SCORING(1) SINGULAR(0.000000000001) HCONVERGE(0,

ABSOLUTE) LCONVERGE(0, ABSOLUTE) PCONVERGE(0.000001, ABSOLUTE)

/FIXED= leeftijd EOGlinks EOGrechts

oor_hart moment_exposure meting straling segment

PRE POST | SSTYPE(3)

/METHOD=REML

/PRINT=SOLUTION TESTCOV

/RANDOM=INTERCEPT segment | SUBJECT(ppn) COVTYPE(VC)

/RANDOM=INTERCEPT segment | SUBJECT(ppn*meting) COVTYPE(VC)

/RANDOM=INTERCEPT segment | SUBJECT(ppn*meting*conditie) COVTYPE(VC)

/REPEATED=segment | SUBJECT(ppn*meting*conditie) COVTYPE(AR1).

COMMENT links-rechts verschil slowbeta.

compute linksrechts_sb = mean_slowbeta_links - mean_slowbeta_rechts.

execute.

USE ALL.

COMPUTE filter_$=(EOGlinks_sum_mean < 10000000 and EOGrechts_sum_mean < 10000000 and straling_valide = 1).

FILTER BY filter_$.

EXECUTE.

MIXED linksrechts_sb WITH PRE POST segment meting leeftijd oor_hart moment_exposure

straling EOGlinks EOGrechts

/CRITERIA=CIN(95) MXITER(100) MXSTEP(10) SCORING(1) SINGULAR(0.000000000001) HCONVERGE(0,

ABSOLUTE) LCONVERGE(0, ABSOLUTE) PCONVERGE(0.000001, ABSOLUTE)

/FIXED= leeftijd EOGlinks EOGrechts

oor_hart moment_exposure meting straling segment

PRE POST | SSTYPE(3)

/METHOD=REML

/PRINT=SOLUTION TESTCOV

/RANDOM=INTERCEPT segment | SUBJECT(ppn) COVTYPE(VC)

/RANDOM=INTERCEPT segment | SUBJECT(ppn*meting) COVTYPE(VC)

/RANDOM=INTERCEPT segment | SUBJECT(ppn*meting*conditie) COVTYPE(VC)

/REPEATED=segment | SUBJECT(ppn*meting*conditie) COVTYPE(AR1).

COMMENT nu over de verschillende regios.

USE ALL.

COMPUTE filter_$=(EOGlinks_sum_mean < 10000000 and EOGrechts_sum_mean < 10000000 and straling_valide = 1).

FILTER BY filter_$.

EXECUTE.

MIXED delta_frontaal WITH PRE POST segment meting leeftijd oor_hart moment_exposure

straling EOGlinks EOGrechts

/CRITERIA=CIN(95) MXITER(100) MXSTEP(10) SCORING(1) SINGULAR(0.000000000001) HCONVERGE(0,

ABSOLUTE) LCONVERGE(0, ABSOLUTE) PCONVERGE(0.000001, ABSOLUTE)

/FIXED= leeftijd EOGlinks EOGrechts

oor_hart moment_exposure meting straling segment

PRE POST | SSTYPE(3)

/METHOD=REML

/PRINT=SOLUTION TESTCOV

/RANDOM=INTERCEPT segment | SUBJECT(ppn) COVTYPE(VC)

/RANDOM=INTERCEPT segment | SUBJECT(ppn*meting) COVTYPE(VC)

/RANDOM=INTERCEPT segment | SUBJECT(ppn*meting*conditie) COVTYPE(VC)

/REPEATED=segment | SUBJECT(ppn*meting*conditie) COVTYPE(AR1).

MIXED delta_centraal WITH PRE POST segment meting leeftijd oor_hart moment_exposure

straling EOGlinks EOGrechts

/CRITERIA=CIN(95) MXITER(100) MXSTEP(10) SCORING(1) SINGULAR(0.000000000001) HCONVERGE(0,

ABSOLUTE) LCONVERGE(0, ABSOLUTE) PCONVERGE(0.000001, ABSOLUTE)

/FIXED= leeftijd EOGlinks EOGrechts

oor_hart moment_exposure meting straling segment

PRE POST | SSTYPE(3)

/METHOD=REML

/PRINT=SOLUTION TESTCOV

/RANDOM=INTERCEPT segment | SUBJECT(ppn) COVTYPE(VC)

/RANDOM=INTERCEPT segment | SUBJECT(ppn*meting) COVTYPE(VC)

/RANDOM=INTERCEPT segment | SUBJECT(ppn*meting*conditie) COVTYPE(VC)

/REPEATED=segment | SUBJECT(ppn*meting*conditie) COVTYPE(AR1).

MIXED delta_parietaal WITH PRE POST segment meting leeftijd oor_hart moment_exposure

straling EOGlinks EOGrechts

/CRITERIA=CIN(95) MXITER(100) MXSTEP(10) SCORING(1) SINGULAR(0.000000000001) HCONVERGE(0,

ABSOLUTE) LCONVERGE(0, ABSOLUTE) PCONVERGE(0.000001, ABSOLUTE)

/FIXED= leeftijd EOGlinks EOGrechts

oor_hart moment_exposure meting straling segment

PRE POST | SSTYPE(3)

/METHOD=REML

/PRINT=SOLUTION TESTCOV

/RANDOM=INTERCEPT segment | SUBJECT(ppn) COVTYPE(VC)

/RANDOM=INTERCEPT segment | SUBJECT(ppn*meting) COVTYPE(VC)

/RANDOM=INTERCEPT segment | SUBJECT(ppn*meting*conditie) COVTYPE(VC)

/REPEATED=segment | SUBJECT(ppn*meting*conditie) COVTYPE(AR1).

MIXED delta_occipitaal WITH PRE POST segment meting leeftijd oor_hart moment_exposure

straling EOGlinks EOGrechts

/CRITERIA=CIN(95) MXITER(100) MXSTEP(10) SCORING(1) SINGULAR(0.000000000001) HCONVERGE(0,

ABSOLUTE) LCONVERGE(0, ABSOLUTE) PCONVERGE(0.000001, ABSOLUTE)

/FIXED= leeftijd EOGlinks EOGrechts

oor_hart moment_exposure meting straling segment

PRE POST | SSTYPE(3)

/METHOD=REML

/PRINT=SOLUTION TESTCOV

/RANDOM=INTERCEPT segment | SUBJECT(ppn) COVTYPE(VC)

/RANDOM=INTERCEPT segment | SUBJECT(ppn*meting) COVTYPE(VC)

/RANDOM=INTERCEPT segment | SUBJECT(ppn*meting*conditie) COVTYPE(VC)

/REPEATED=segment | SUBJECT(ppn*meting*conditie) COVTYPE(AR1).

MIXED theta_frontaal WITH PRE POST segment meting leeftijd oor_hart moment_exposure

straling EOGlinks EOGrechts

/CRITERIA=CIN(95) MXITER(100) MXSTEP(10) SCORING(1) SINGULAR(0.000000000001) HCONVERGE(0,

ABSOLUTE) LCONVERGE(0, ABSOLUTE) PCONVERGE(0.000001, ABSOLUTE)

/FIXED= leeftijd EOGlinks EOGrechts

oor_hart moment_exposure meting straling segment

PRE POST | SSTYPE(3)

/METHOD=REML

/PRINT=SOLUTION TESTCOV

/RANDOM=INTERCEPT segment | SUBJECT(ppn) COVTYPE(VC)

/RANDOM=INTERCEPT segment | SUBJECT(ppn*meting) COVTYPE(VC)

/RANDOM=INTERCEPT segment | SUBJECT(ppn*meting*conditie) COVTYPE(VC)

/REPEATED=segment | SUBJECT(ppn*meting*conditie) COVTYPE(AR1).

MIXED theta_centraal WITH PRE POST segment meting leeftijd oor_hart moment_exposure

straling EOGlinks EOGrechts

/CRITERIA=CIN(95) MXITER(100) MXSTEP(10) SCORING(1) SINGULAR(0.000000000001) HCONVERGE(0,

ABSOLUTE) LCONVERGE(0, ABSOLUTE) PCONVERGE(0.000001, ABSOLUTE)

/FIXED= leeftijd EOGlinks EOGrechts

oor_hart moment_exposure meting straling segment

PRE POST | SSTYPE(3)

/METHOD=REML

/PRINT=SOLUTION TESTCOV

/RANDOM=INTERCEPT segment | SUBJECT(ppn) COVTYPE(VC)

/RANDOM=INTERCEPT segment | SUBJECT(ppn*meting) COVTYPE(VC)

/RANDOM=INTERCEPT segment | SUBJECT(ppn*meting*conditie) COVTYPE(VC)

/REPEATED=segment | SUBJECT(ppn*meting*conditie) COVTYPE(AR1).

MIXED theta_parietaal WITH PRE POST segment meting leeftijd oor_hart moment_exposure

straling EOGlinks EOGrechts

/CRITERIA=CIN(95) MXITER(100) MXSTEP(10) SCORING(1) SINGULAR(0.000000000001) HCONVERGE(0,

ABSOLUTE) LCONVERGE(0, ABSOLUTE) PCONVERGE(0.000001, ABSOLUTE)

/FIXED= leeftijd EOGlinks EOGrechts

oor_hart moment_exposure meting straling segment

PRE POST | SSTYPE(3)

/METHOD=REML

/PRINT=SOLUTION TESTCOV

/RANDOM=INTERCEPT segment | SUBJECT(ppn) COVTYPE(VC)

/RANDOM=INTERCEPT segment | SUBJECT(ppn*meting) COVTYPE(VC)

/RANDOM=INTERCEPT segment | SUBJECT(ppn*meting*conditie) COVTYPE(VC)

/REPEATED=segment | SUBJECT(ppn*meting*conditie) COVTYPE(AR1).

MIXED theta_occipitaal WITH PRE POST segment meting leeftijd oor_hart moment_exposure

straling EOGlinks EOGrechts

/CRITERIA=CIN(95) MXITER(100) MXSTEP(10) SCORING(1) SINGULAR(0.000000000001) HCONVERGE(0,

ABSOLUTE) LCONVERGE(0, ABSOLUTE) PCONVERGE(0.000001, ABSOLUTE)

/FIXED= leeftijd EOGlinks EOGrechts

oor_hart moment_exposure meting straling segment

PRE POST | SSTYPE(3)

/METHOD=REML

/PRINT=SOLUTION TESTCOV

/RANDOM=INTERCEPT segment | SUBJECT(ppn) COVTYPE(VC)

/RANDOM=INTERCEPT segment | SUBJECT(ppn*meting) COVTYPE(VC)

/RANDOM=INTERCEPT segment | SUBJECT(ppn*meting*conditie) COVTYPE(VC)

/REPEATED=segment | SUBJECT(ppn*meting*conditie) COVTYPE(AR1).

MIXED alpha_frontaal WITH PRE POST segment meting leeftijd oor_hart moment_exposure

straling EOGlinks EOGrechts

/CRITERIA=CIN(95) MXITER(100) MXSTEP(10) SCORING(1) SINGULAR(0.000000000001) HCONVERGE(0,

ABSOLUTE) LCONVERGE(0, ABSOLUTE) PCONVERGE(0.000001, ABSOLUTE)

/FIXED= leeftijd EOGlinks EOGrechts

oor_hart moment_exposure meting straling segment

PRE POST | SSTYPE(3)

/METHOD=REML

/PRINT=SOLUTION TESTCOV

/RANDOM=INTERCEPT segment | SUBJECT(ppn) COVTYPE(VC)

/RANDOM=INTERCEPT segment | SUBJECT(ppn*meting) COVTYPE(VC)

/RANDOM=INTERCEPT segment | SUBJECT(ppn*meting*conditie) COVTYPE(VC)

/REPEATED=segment | SUBJECT(ppn*meting*conditie) COVTYPE(AR1).

MIXED alpha_centraal WITH PRE POST segment meting leeftijd oor_hart moment_exposure

straling EOGlinks EOGrechts

/CRITERIA=CIN(95) MXITER(100) MXSTEP(10) SCORING(1) SINGULAR(0.000000000001) HCONVERGE(0,

ABSOLUTE) LCONVERGE(0, ABSOLUTE) PCONVERGE(0.000001, ABSOLUTE)

/FIXED= leeftijd EOGlinks EOGrechts

oor_hart moment_exposure meting straling segment

PRE POST | SSTYPE(3)

/METHOD=REML

/PRINT=SOLUTION TESTCOV

/RANDOM=INTERCEPT segment | SUBJECT(ppn) COVTYPE(VC)

/RANDOM=INTERCEPT segment | SUBJECT(ppn*meting) COVTYPE(VC)

/RANDOM=INTERCEPT segment | SUBJECT(ppn*meting*conditie) COVTYPE(VC)

/REPEATED=segment | SUBJECT(ppn*meting*conditie) COVTYPE(AR1).

MIXED alpha_parietaal WITH PRE POST segment meting leeftijd oor_hart moment_exposure

straling EOGlinks EOGrechts

/CRITERIA=CIN(95) MXITER(100) MXSTEP(10) SCORING(1) SINGULAR(0.000000000001) HCONVERGE(0,

ABSOLUTE) LCONVERGE(0, ABSOLUTE) PCONVERGE(0.000001, ABSOLUTE)

/FIXED= leeftijd EOGlinks EOGrechts

oor_hart moment_exposure meting straling segment

PRE POST | SSTYPE(3)

/METHOD=REML

/PRINT=SOLUTION TESTCOV

/RANDOM=INTERCEPT segment | SUBJECT(ppn) COVTYPE(VC)

/RANDOM=INTERCEPT segment | SUBJECT(ppn*meting) COVTYPE(VC)

/RANDOM=INTERCEPT segment | SUBJECT(ppn*meting*conditie) COVTYPE(VC)

/REPEATED=segment | SUBJECT(ppn*meting*conditie) COVTYPE(AR1).

MIXED alpha_occipitaal WITH PRE POST segment meting leeftijd oor_hart moment_exposure

straling EOGlinks EOGrechts

/CRITERIA=CIN(95) MXITER(100) MXSTEP(10) SCORING(1) SINGULAR(0.000000000001) HCONVERGE(0,

ABSOLUTE) LCONVERGE(0, ABSOLUTE) PCONVERGE(0.000001, ABSOLUTE)

/FIXED= leeftijd EOGlinks EOGrechts

oor_hart moment_exposure meting straling segment

PRE POST | SSTYPE(3)

/METHOD=REML

/PRINT=SOLUTION TESTCOV

/RANDOM=INTERCEPT segment | SUBJECT(ppn) COVTYPE(VC)

/RANDOM=INTERCEPT segment | SUBJECT(ppn*meting) COVTYPE(VC)

/RANDOM=INTERCEPT segment | SUBJECT(ppn*meting*conditie) COVTYPE(VC)

/REPEATED=segment | SUBJECT(ppn*meting*conditie) COVTYPE(AR1).

MIXED slowbeta_frontaal WITH PRE POST segment meting leeftijd oor_hart moment_exposure

straling EOGlinks EOGrechts

/CRITERIA=CIN(95) MXITER(100) MXSTEP(10) SCORING(1) SINGULAR(0.000000000001) HCONVERGE(0,

ABSOLUTE) LCONVERGE(0, ABSOLUTE) PCONVERGE(0.000001, ABSOLUTE)

/FIXED= leeftijd EOGlinks EOGrechts

oor_hart moment_exposure meting straling segment

PRE POST | SSTYPE(3)

/METHOD=REML

/PRINT=SOLUTION TESTCOV

/RANDOM=INTERCEPT segment | SUBJECT(ppn) COVTYPE(VC)

/RANDOM=INTERCEPT segment | SUBJECT(ppn*meting) COVTYPE(VC)

/RANDOM=INTERCEPT segment | SUBJECT(ppn*meting*conditie) COVTYPE(VC)

/REPEATED=segment | SUBJECT(ppn*meting*conditie) COVTYPE(AR1).

MIXED slowbeta_centraal WITH PRE POST segment meting leeftijd oor_hart moment_exposure

straling EOGlinks EOGrechts

/CRITERIA=CIN(95) MXITER(100) MXSTEP(10) SCORING(1) SINGULAR(0.000000000001) HCONVERGE(0,

ABSOLUTE) LCONVERGE(0, ABSOLUTE) PCONVERGE(0.000001, ABSOLUTE)

/FIXED= leeftijd EOGlinks EOGrechts

oor_hart moment_exposure meting straling segment

PRE POST | SSTYPE(3)

/METHOD=REML

/PRINT=SOLUTION TESTCOV

/RANDOM=INTERCEPT segment | SUBJECT(ppn) COVTYPE(VC)

/RANDOM=INTERCEPT segment | SUBJECT(ppn*meting) COVTYPE(VC)

/RANDOM=INTERCEPT segment | SUBJECT(ppn*meting*conditie) COVTYPE(VC)

/REPEATED=segment | SUBJECT(ppn*meting*conditie) COVTYPE(AR1).

MIXED slowbeta_parietaal WITH PRE POST segment meting leeftijd oor_hart moment_exposure

straling EOGlinks EOGrechts

/CRITERIA=CIN(95) MXITER(100) MXSTEP(10) SCORING(1) SINGULAR(0.000000000001) HCONVERGE(0,

ABSOLUTE) LCONVERGE(0, ABSOLUTE) PCONVERGE(0.000001, ABSOLUTE)

/FIXED= leeftijd EOGlinks EOGrechts

oor_hart moment_exposure meting straling segment

PRE POST | SSTYPE(3)

/METHOD=REML

/PRINT=SOLUTION TESTCOV

/RANDOM=INTERCEPT segment | SUBJECT(ppn) COVTYPE(VC)

/RANDOM=INTERCEPT segment | SUBJECT(ppn*meting) COVTYPE(VC)

/RANDOM=INTERCEPT segment | SUBJECT(ppn*meting*conditie) COVTYPE(VC)

/REPEATED=segment | SUBJECT(ppn*meting*conditie) COVTYPE(AR1).

MIXED slowbeta_occipitaal WITH PRE POST segment meting leeftijd oor_hart moment_exposure

straling EOGlinks EOGrechts

/CRITERIA=CIN(95) MXITER(100) MXSTEP(10) SCORING(1) SINGULAR(0.000000000001) HCONVERGE(0,

ABSOLUTE) LCONVERGE(0, ABSOLUTE) PCONVERGE(0.000001, ABSOLUTE)

/FIXED= leeftijd EOGlinks EOGrechts

oor_hart moment_exposure meting straling segment

PRE POST | SSTYPE(3)

/METHOD=REML

/PRINT=SOLUTION TESTCOV

/RANDOM=INTERCEPT segment | SUBJECT(ppn) COVTYPE(VC)

/RANDOM=INTERCEPT segment | SUBJECT(ppn*meting) COVTYPE(VC)

/RANDOM=INTERCEPT segment | SUBJECT(ppn*meting*conditie) COVTYPE(VC)

/REPEATED=segment | SUBJECT(ppn*meting*conditie) COVTYPE(AR1).

MIXED fastbeta_frontaal WITH PRE POST segment meting leeftijd oor_hart moment_exposure

straling EOGlinks EOGrechts

/CRITERIA=CIN(95) MXITER(100) MXSTEP(10) SCORING(1) SINGULAR(0.000000000001) HCONVERGE(0,

ABSOLUTE) LCONVERGE(0, ABSOLUTE) PCONVERGE(0.000001, ABSOLUTE)

/FIXED= leeftijd EOGlinks EOGrechts

oor_hart moment_exposure meting straling segment

PRE POST | SSTYPE(3)

/METHOD=REML

/PRINT=SOLUTION TESTCOV

/RANDOM=INTERCEPT segment | SUBJECT(ppn) COVTYPE(VC)

/RANDOM=INTERCEPT segment | SUBJECT(ppn*meting) COVTYPE(VC)

/RANDOM=INTERCEPT segment | SUBJECT(ppn*meting*conditie) COVTYPE(VC)

/REPEATED=segment | SUBJECT(ppn*meting*conditie) COVTYPE(AR1).

MIXED fastbeta_centraal WITH PRE POST segment meting leeftijd oor_hart moment_exposure

straling EOGlinks EOGrechts

/CRITERIA=CIN(95) MXITER(100) MXSTEP(10) SCORING(1) SINGULAR(0.000000000001) HCONVERGE(0,

ABSOLUTE) LCONVERGE(0, ABSOLUTE) PCONVERGE(0.000001, ABSOLUTE)

/FIXED= leeftijd EOGlinks EOGrechts

oor_hart moment_exposure meting straling segment

PRE POST | SSTYPE(3)

/METHOD=REML

/PRINT=SOLUTION TESTCOV

/RANDOM=INTERCEPT segment | SUBJECT(ppn) COVTYPE(VC)

/RANDOM=INTERCEPT segment | SUBJECT(ppn*meting) COVTYPE(VC)

/RANDOM=INTERCEPT segment | SUBJECT(ppn*meting*conditie) COVTYPE(VC)

/REPEATED=segment | SUBJECT(ppn*meting*conditie) COVTYPE(AR1).

MIXED fastbeta_parietaal WITH PRE POST segment meting leeftijd oor_hart moment_exposure

straling EOGlinks EOGrechts

/CRITERIA=CIN(95) MXITER(100) MXSTEP(10) SCORING(1) SINGULAR(0.000000000001) HCONVERGE(0,

ABSOLUTE) LCONVERGE(0, ABSOLUTE) PCONVERGE(0.000001, ABSOLUTE)

/FIXED= leeftijd EOGlinks EOGrechts

oor_hart moment_exposure meting straling segment

PRE POST | SSTYPE(3)

/METHOD=REML

/PRINT=SOLUTION TESTCOV

/RANDOM=INTERCEPT segment | SUBJECT(ppn) COVTYPE(VC)

/RANDOM=INTERCEPT segment | SUBJECT(ppn*meting) COVTYPE(VC)

/RANDOM=INTERCEPT segment | SUBJECT(ppn*meting*conditie) COVTYPE(VC)

/REPEATED=segment | SUBJECT(ppn*meting*conditie) COVTYPE(AR1).

MIXED fastbeta_occipitaal WITH PRE POST segment meting leeftijd oor_hart moment_exposure

straling EOGlinks EOGrechts

/CRITERIA=CIN(95) MXITER(100) MXSTEP(10) SCORING(1) SINGULAR(0.000000000001) HCONVERGE(0,

ABSOLUTE) LCONVERGE(0, ABSOLUTE) PCONVERGE(0.000001, ABSOLUTE)

/FIXED= leeftijd EOGlinks EOGrechts

oor_hart moment_exposure meting straling segment

PRE POST | SSTYPE(3)

/METHOD=REML

/PRINT=SOLUTION TESTCOV

/RANDOM=INTERCEPT segment | SUBJECT(ppn) COVTYPE(VC)

/RANDOM=INTERCEPT segment | SUBJECT(ppn*meting) COVTYPE(VC)

/RANDOM=INTERCEPT segment | SUBJECT(ppn*meting*conditie) COVTYPE(VC)

/REPEATED=segment | SUBJECT(ppn*meting*conditie) COVTYPE(AR1).

MIXED gamma_frontaal WITH PRE POST segment meting leeftijd oor_hart moment_exposure

straling EOGlinks EOGrechts

/CRITERIA=CIN(95) MXITER(100) MXSTEP(10) SCORING(1) SINGULAR(0.000000000001) HCONVERGE(0,

ABSOLUTE) LCONVERGE(0, ABSOLUTE) PCONVERGE(0.000001, ABSOLUTE)

/FIXED= leeftijd EOGlinks EOGrechts

oor_hart moment_exposure meting straling segment

PRE POST | SSTYPE(3)

/METHOD=REML

/PRINT=SOLUTION TESTCOV

/RANDOM=INTERCEPT segment | SUBJECT(ppn) COVTYPE(VC)

/RANDOM=INTERCEPT segment | SUBJECT(ppn*meting) COVTYPE(VC)

/RANDOM=INTERCEPT segment | SUBJECT(ppn*meting*conditie) COVTYPE(VC)

/REPEATED=segment | SUBJECT(ppn*meting*conditie) COVTYPE(AR1).

MIXED gamma_centraal WITH PRE POST segment meting leeftijd oor_hart moment_exposure

straling EOGlinks EOGrechts

/CRITERIA=CIN(95) MXITER(100) MXSTEP(10) SCORING(1) SINGULAR(0.000000000001) HCONVERGE(0,

ABSOLUTE) LCONVERGE(0, ABSOLUTE) PCONVERGE(0.000001, ABSOLUTE)

/FIXED= leeftijd EOGlinks EOGrechts

oor_hart moment_exposure meting straling segment

PRE POST | SSTYPE(3)

/METHOD=REML

/PRINT=SOLUTION TESTCOV

/RANDOM=INTERCEPT segment | SUBJECT(ppn) COVTYPE(VC)

/RANDOM=INTERCEPT segment | SUBJECT(ppn*meting) COVTYPE(VC)

/RANDOM=INTERCEPT segment | SUBJECT(ppn*meting*conditie) COVTYPE(VC)

/REPEATED=segment | SUBJECT(ppn*meting*conditie) COVTYPE(AR1).

MIXED gamma_parietaal WITH PRE POST segment meting leeftijd oor_hart moment_exposure

straling EOGlinks EOGrechts

/CRITERIA=CIN(95) MXITER(100) MXSTEP(10) SCORING(1) SINGULAR(0.000000000001) HCONVERGE(0,

ABSOLUTE) LCONVERGE(0, ABSOLUTE) PCONVERGE(0.000001, ABSOLUTE)

/FIXED= leeftijd EOGlinks EOGrechts

oor_hart moment_exposure meting straling segment

PRE POST | SSTYPE(3)

/METHOD=REML

/PRINT=SOLUTION TESTCOV

/RANDOM=INTERCEPT segment | SUBJECT(ppn) COVTYPE(VC)

/RANDOM=INTERCEPT segment | SUBJECT(ppn*meting) COVTYPE(VC)

/RANDOM=INTERCEPT segment | SUBJECT(ppn*meting*conditie) COVTYPE(VC)

/REPEATED=segment | SUBJECT(ppn*meting*conditie) COVTYPE(AR1).

MIXED gamma_occipitaal WITH PRE POST segment meting leeftijd oor_hart moment_exposure

straling EOGlinks EOGrechts

/CRITERIA=CIN(95) MXITER(100) MXSTEP(10) SCORING(1) SINGULAR(0.000000000001) HCONVERGE(0,

ABSOLUTE) LCONVERGE(0, ABSOLUTE) PCONVERGE(0.000001, ABSOLUTE)

/FIXED= leeftijd EOGlinks EOGrechts

oor_hart moment_exposure meting straling segment

PRE POST | SSTYPE(3)

/METHOD=REML

/PRINT=SOLUTION TESTCOV

/RANDOM=INTERCEPT segment | SUBJECT(ppn) COVTYPE(VC)

/RANDOM=INTERCEPT segment | SUBJECT(ppn*meting) COVTYPE(VC)

/RANDOM=INTERCEPT segment | SUBJECT(ppn*meting*conditie) COVTYPE(VC)

/REPEATED=segment | SUBJECT(ppn*meting*conditie) COVTYPE(AR1).
